# Postnatal Care Utilisation Among Adolescent Mothers in India: A Pooled Cross‐Sectional Study of NFHS‐4 and NFHS‐5

**DOI:** 10.1002/puh2.70298

**Published:** 2026-06-12

**Authors:** Anshika Singh, Aditya Singh, Rakesh Chandra, Muhammad Hossain, Utkarsh Jain

**Affiliations:** ^1^ Banaras Hindu University Varanasi India; ^2^ Tata Institute of Social Sciences Mumbai India; ^3^ Birmingham City University Birmingham UK

**Keywords:** adolescent mothers, India, maternal health, NFHS‐4, NFHS‐5, postnatal care

## Abstract

Although postnatal care (PNC) is a critical component of maternal health services, empirical evidence on its utilisation and associated factors among adolescent mothers in India remains limited. Using nationally representative data from the fourth (2015–16) and fifth (2019–21) rounds of the National Family Health Survey, this study examines trends and determinants of PNC utilisation among mothers aged 15–19 years. PNC within 42 days of delivery was analysed as a binary outcome using multivariable logistic regression. PNC utilisation among adolescent mothers increased substantially between the two survey rounds, from 69.9% in 2015–16 to 82.3% in 2019–21. Despite this progress, marked socio‐economic and regional inequalities persisted. By 2019–21, the lowest utilisation was concentrated in the north‐eastern region, particularly in Nagaland (47.1%), Mizoram (64.7%), Assam (69.3%), and Arunachal Pradesh (69.7%). Multivariable regression analysis showed that PNC utilisation was most strongly associated with place of delivery and antenatal care attendance, with a clear dose–response relationship for the number of antenatal care visits. Adolescent mothers delivering in health facilities and those receiving eight or more antenatal care visits were significantly more likely to utilise PNC. Household wealth, health insurance coverage, and region of residence were also significant predictors. Although India has made substantial progress in antenatal care and institutional delivery, PNC utilisation among adolescent mothers remains uneven. Improving continuity between antenatal care, delivery services and postnatal follow‐up, and addressing persistent socio‐economic and regional inequalities, is important for ensuring equitable access to PNC.

## Graphical abstract

Postnatal care utilisation among adolescent mothers in India increased substantially between NFHS‐4 and NFHS‐5. However, significant socio‐economic and regional inequalities persist, with antenatal care utilisation and facility delivery strongly predicting postnatal care uptake, highlighting the importance of strengthening continuity of maternal healthcare.

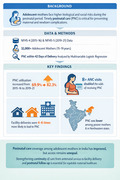

## 1 Introduction

The postnatal period (the first 6 weeks following childbirth) is one of the most critical yet comparatively neglected phases within the continuum of maternal and newborn care [1]. A substantial proportion of maternal deaths, severe morbidities and neonatal complications occur during this period, particularly within the first 48 h after delivery. Timely postnatal care (PNC) enables early identification and management of postpartum haemorrhage, hypertensive disorders, sepsis and anaemia [2] while also providing essential services such as breastfeeding counselling, contraception advice, nutritional guidance, immunisation linkage and newborn health assessment [3]. Despite global progress in expanding antenatal care and institutional delivery, PNC has often lagged behind in coverage and quality, reflecting a programmatic emphasis on childbirth over sustained postpartum follow‐up [4].

Within this broader context, adolescent motherhood represents a distinct public health and equity concern [5]. Adolescents, defined by the World Health Organization as individuals aged 10–19 years [6], comprise approximately one‐fifth of India's population [7]. Although adolescent childbearing in India has declined steadily over the past two decades, early motherhood remains a persistent reality. According to the fifth round of the National Family Health Survey (NFHS) (2019–21), approximately 6.8% of women aged 15–19 years in India have begun childbearing, either having already given birth or being pregnant at the time of the survey [8]. Although this represents a substantial decline from 16% in NFHS‐3 (2005–06) and 7.9% in NFHS‐4 (2015–16), early motherhood remains concentrated in socioeconomically disadvantaged and rural populations, with considerable variation across states. However, these national averages conceal substantial socio‐economic and geographic disparities, with higher prevalence observed among poorer households, rural populations, and in several central and eastern states.

Adolescent pregnancy is associated with well‐documented biological risks [9]. Compared with women in their twenties, adolescent mothers face higher risks of hypertensive disorders, puerperal infections, obstructed labour and anaemia. Their infants are more likely to be born preterm, have low birth weight, and experience neonatal morbidity or mortality [10]. However, biological vulnerability represents only one dimension of disadvantage. Adolescent mothers often occupy relatively marginal positions within household and community structures. Early marriage, lower levels of education, financial dependence, restricted mobility and limited decision‐making autonomy may reduce their ability to recognise potential health complications, seek care independently or access health services in a timely manner [11]. These overlapping social and structural conditions suggest that age should not be viewed simply as a demographic characteristic, but as a reflection of broader disadvantages that shape maternal healthcare utilisation. Given these biological and social vulnerabilities, timely PNC is particularly important for adolescent mothers.

National evidence indicates that although PNC coverage in India has improved, it remains incomplete and unequally distributed. NFHS‐5 reports that approximately 83% of women who gave birth in the 5 years preceding the survey received a postnatal health check within 6 weeks of delivery, meaning that about 17% did not receive any PNC [8]. More importantly, timely care during the most critical period immediately after childbirth remains limited. Only about half of mothers received PNC within the first 24 h after birth, despite the World Health Organization's recommendation that all mothers receive PNC during this period. Disparities in PNC utilisation persist across wealth, education, residence and place of delivery, suggesting that improvements in institutional delivery have not necessarily ensured continuity of care after childbirth [8]. These gaps also reflect broader health system constraints, including uneven availability of postnatal follow‐up services, variation in frontline health worker outreach and regional differences in health system capacity. Age‐based differences are also evident. NFHS‐5 data indicate that nearly one in five adolescent mothers (aged <20 years at birth) did not receive any PNC within 42 days of delivery, compared with about one in six women aged 20–34 years [8]. Although this absolute gap appears modest at the national level, it may nonetheless be programmatically important for adolescent mothers, given their heightened biological vulnerability and comparatively limited autonomy in healthcare decision‐making, which may amplify the consequences of missed postpartum care.

A substantial body of literature has examined the determinants and inequities in PNC utilisation in India [12, 13, 14, 15] and other low‐ and middle‐income countries [16, 17]. Analyses based on nationally representative surveys, including successive rounds of NFHS, have consistently identified socio‐economic status, educational attainment, place of delivery, rural–urban residence and caste as key predictors of PNC uptake among women overall [14, 15, 18, 19, 20, 21, 22]. More recent studies have also explored inequalities in the completion of the maternal continuum of care, highlighting that improvements in institutional delivery have not necessarily translated into timely postpartum follow‐up [20, 21, 23, 24]. Geospatial and multilevel investigations have further documented marked regional disparities and demonstrated how community‐level factors, such as access to health facilities, frontline health worker coverage and exposure to environmental or contextual shocks, interact with individual vulnerabilities to shape patterns of service utilisation [13, 25, 26].

Despite the growing body of research on maternal healthcare utilisation, literature examining PNC utilisation specifically among adolescent mothers remains limited. Most studies assess women of reproductive age collectively or focus on overall continuum‐of‐care indicators without specifically disaggregating PNC by age. Consequently, it remains unclear whether the determinants and inequalities identified for women overall apply similarly or more strongly to adolescents aged 15–19 years. This gap is particularly relevant in India, where adolescent pregnancies are concentrated in states and social groups that already experience lower levels of PNC coverage. In a context where institutional delivery has expanded but postpartum follow‐up remains uneven, separate examination of adolescent mothers is necessary to better understand their specific vulnerabilities and to inform more responsive maternal health policies.

This study addresses this gap by using nationally representative data from the NFHS rounds 4 (2015–16) and 5 (2019–21) to examine PNC utilisation among adolescent mothers in India. Drawing on a pooled sample of more than 32,000 adolescent mothers, the analysis examines changes between survey rounds and identifies the socio‐economic, demographic and geographic factors associated with receiving PNC within the recommended 6‐week postpartum period. By examining adolescents as a distinct analytical group, the study explores whether recent improvements in PNC utilisation have been shared across socio‐economic and regional groups. It also assesses how PNC utilisation varies according to household wealth, health insurance coverage, antenatal care attendance, place of delivery and region of residence. The findings contribute to understanding how age intersects with structural inequalities in shaping postpartum service utilisation. By documenting both progress and remaining gaps, the study provides evidence to inform adolescent‐responsive strategies within India's maternal health framework and to strengthen the continuum of care beyond childbirth.

## 2 Data and Methods

This study draws on data from the fourth and fifth rounds of NFHS, conducted in 2015–16 (NFHS‐4) and 2019–21 (NFHS‐5). The NFHS‐4 interviewed 699,686 women, and the NFHS‐5 interviewed 724,115 women, all aged 15–49 years. The survey is a particularly rich source for analysing reproductive and maternal health, as it collects detailed information on reproductive history, menstrual hygiene, family planning, maternal health, marital and sexual behaviour, fertility preferences, domestic violence and a wide range of health indicators. The survey follows a two‐stage stratified sampling design, with further details available in the NFHS reports [8, 27]. Response rates were high, with 97% of eligible women participating in both rounds.

**FIGURE 1:**
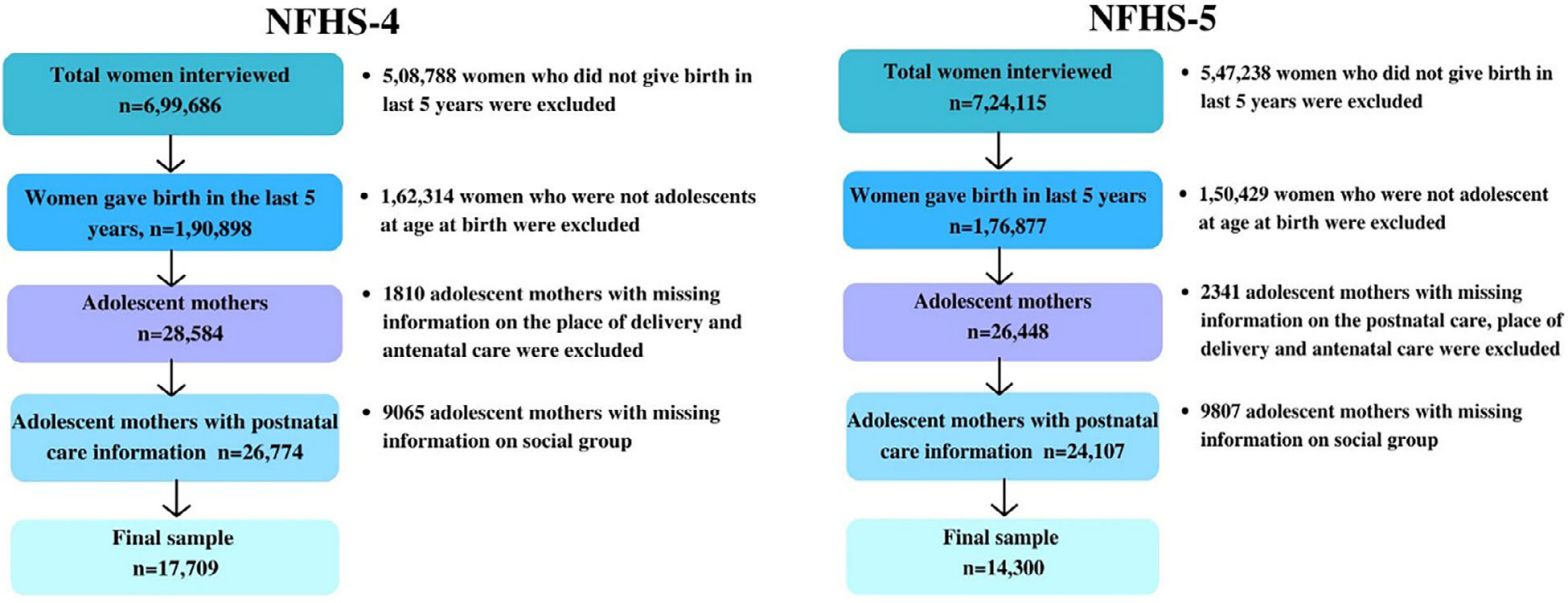
Flow diagram showing sample selection for the pooled analysis of adolescent mothers from NFHS‐4 and NFHS‐5. NFHS, National Family Health Survey.

For the purposes of this study, women who had not given birth in the 5 years preceding the survey were excluded. Among those remaining, we further restricted the sample to mothers who were aged 15–19 at the time of their most recent birth. This yielded an analytical sample of 17,709 adolescent mothers from NFHS‐4 and 14,300 from NFHS‐5. The sample selection process is summarised in Figure 1. Although the World Health Organization defines adolescents as individuals aged 10–19 years, this study focuses on late adolescence (15–19 years) because information on PNC in the NFHS is collected only for women aged 15–49 years who had a recent birth.

### 2.1 Dependent Variable

The outcome variable in this study is the utilisation of PNC within 42 days of delivery. Women were asked to report the timing of their first PNC, with responses recorded in continuous form. These responses were then recoded into a binary outcome: those who received PNC between delivery and the 42nd day (i.e., within 6 weeks) were classified as having ‘received postnatal care’ (coded as 1) and otherwise (coded as 0). This measure captures whether any PNC was received within 42 days of delivery and does not distinguish the timing, number or quality of postnatal visits.

### 2.2 Independent Variables

The selection of independent variables was guided by Andersen's behavioural model of health service utilisation, which conceptualises healthcare use as a function of predisposing, enabling and need‐related factors. On the basis of this framework and prior empirical studies, PNC utilisation was examined in relation to biodemographic, geographic and socio‐economic characteristics, including marital status, respondent's education, social group, religion, household wealth, health insurance coverage, utilisation of antenatal care, sex of the child, birth order, place of delivery, residence and region [12, 22, 28]. The predictor variables included in the study, along with their definitions and categories, are presented in Table 1.

**TABLE 1:** Operational definition of variables used in the study.

Variables	Description
Current marital status	Respondents were asked about their current marital status. Those who reported being currently married were coded as 0, whereas those who were not in union, widowed, separated or divorced were grouped as ‘Not currently married’ and coded as 1
Level of education	Respondents were asked about their highest level of schooling. Education was categorised into four groups: No education (0), Primary (1), Secondary (2) and Higher (3)
Social groups	Social group was categorised into four official categories: Scheduled Caste (1), Scheduled Tribe (2), Other Backward Class (3) and others (0)
Religion	Religion was categorised as Hindu (0), Muslim (1) and Others (2), which includes Christian, Sikh, Buddhist/Neo‐Buddhist, Jain, Jewish, Parsi/Zoroastrian, no religion and other religions
Household wealth index	The wealth index is a composite measure of household socio‐economic status based on asset ownership and housing characteristics. It was categorised into five quintiles: Poorest (0), Poorer (1), Middle (2), Richer (3) and Richest (4)
Number of antenatal care visits	Women were asked about the number of antenatal care visits received during pregnancy. The responses were recorded in continuous form and categorised as Less than four visits (0), Four to seven visits (1) and Eight or more visits (2)
Health insurance coverage	Women were asked whether they were covered under any health insurance scheme. Responses were categorised as No (0) and Yes (1)
Birth order	Women were asked about the birth order of their most recent child. Birth order was categorised as First (0), Second (1) and Third or higher (2)
Place of delivery	Women were asked about the place of their most recent delivery. Deliveries occurring at home (own home, parents’ home or another person's home) were classified as home births (0). Deliveries at government hospitals or other public health facilities were classified as public facility births (1), whereas deliveries at private hospitals, maternity homes, clinics, NGO‐run facilities or other private institutions were classified as private facility births (2)
Place of residence	Place of residence was categorised as Urban (0) and Rural (1)
Region	The regional variable was constructed by grouping states into six regions following the NFHS classification: North (1)—Jammu and Kashmir, Ladakh, Himachal Pradesh, Uttarakhand, Punjab, Chandigarh, Haryana, Delhi and Rajasthan; North‐east (2)—Assam, Arunachal Pradesh, Meghalaya, Nagaland, Tripura, Mizoram, Manipur and Sikkim; Central (3)—Uttar Pradesh, Madhya Pradesh, and Chhattisgarh; West (4)—Gujarat, Daman and Diu, Dadra and Nagar Haveli, Maharashtra and Goa; East (5)—Jharkhand, Bihar, West Bengal and Odisha; and South (6)—Andhra Pradesh, Telangana, Karnataka, Tamil Nadu, Kerala, Andaman and Nicobar Islands, Lakshadweep and Puducherry
Survey year	Survey year indicates the NFHS round used in the analysis. NFHS‐4 (2015–16) was coded as 0 and NFHS‐5 (2019–21) as 1

### 2.3 Statistical Analysis

Descriptive statistics were used to summarise the background characteristics of adolescent mothers and the proportion receiving PNC within 42 days of delivery. Bivariate associations between PNC utilisation and the explanatory variables were examined using Pearson's chi‐square tests.

To identify factors independently associated with PNC utilisation, multivariable logistic regression models were fitted with PNC (yes/no) as the binary outcome. Adjusted odds ratios (AORs) with 95% confidence intervals (CIs) are reported. For the pooled analysis, a survey round indicator (NFHS‐4 vs. NFHS‐5) was included to capture temporal change in PNC utilisation while simultaneously adjusting for individual‐ and household‐level covariates (predisposing, enabling and need‐related factors) guided by Andersen's behavioural model of health service utilisation. All explanatory variables were entered simultaneously in the adjusted model. All analyses accounted for the complex sampling design of the NFHS by applying sampling weights and adjusting for clustering and stratification using the survey (svy) design. The datasets from two NFHS rounds were pooled, and survey year was included as a control variable in the regression models. Multicollinearity among explanatory variables was assessed using the variance inflation factor (VIF); all values were below 2.4, indicating minimal multicollinearity. Statistical significance was evaluated at *p* < 0.05. All analyses were performed using Stata/MP version 16.

## 3 Results

### 3.1 Background Characteristics

Most adolescent mothers were married in both survey rounds. Educational attainment improved over time, with the proportion completing at least secondary education increasing from 62.6% in 2015–16 to 71.2% in 2019–21. The majority of respondents identified as Hindu (around 82%), and a substantial share belonged to Scheduled Castes or Other Backward Classes. Socio‐economic disadvantage remained pronounced, with nearly half of adolescent mothers concentrated in the poorest and poorer wealth quintiles, whereas only about 8% belonged to the richest quintile in both periods. Health insurance coverage was limited, with more than four‐fifths of adolescent mothers reporting no coverage in both rounds. Utilisation of adequate antenatal care remained low, as fewer than one‐fifth received eight or more ANC visits. In contrast, institutional deliveries in public health facilities increased from about 60% in 2015–16 to 70% in 2019–21. Most births to adolescent mothers were first‐order births, and the majority of respondents resided in rural areas (around 78%–79%), with a large concentration in the eastern region of the country.

### 3.2 Utilisation of PNC Among Adolescent Mothers

PNC utilisation among adolescent mothers increased between the two survey rounds, rising from 69.9% in 2015–16 (NFHS‐4) to 82.3% in 2019–21 (NFHS‐5). A similar pattern was observed in state‐wise PNC utilisation among adolescent mothers; however, notable disparities persist across states. The highest utilisation rates were recorded in Goa (100% in both 2015–16 and 2019–21), Tamil Nadu (88.7% in 2015–16 and 98.6% in 2019–21), Kerala (87.2% and 96.5%, respectively), and Odisha (85.9% and 94.7%, respectively). In contrast, Punjab, which had over 90% utilisation in 2015–16, experienced a decline to 84.2% in 2019–21. The lowest utilisation was observed in Nagaland, with only 18.5% in 2015–16 and 47.1% in 2019–21. In 2015–16, other states with low PNC utilisation (besides Nagaland) included Arunachal Pradesh (36.6%), Bihar (52.1%), Jharkhand (52.5%), Uttarakhand (55.8%) and Madhya Pradesh (56.9%).

**FIGURE 2:**
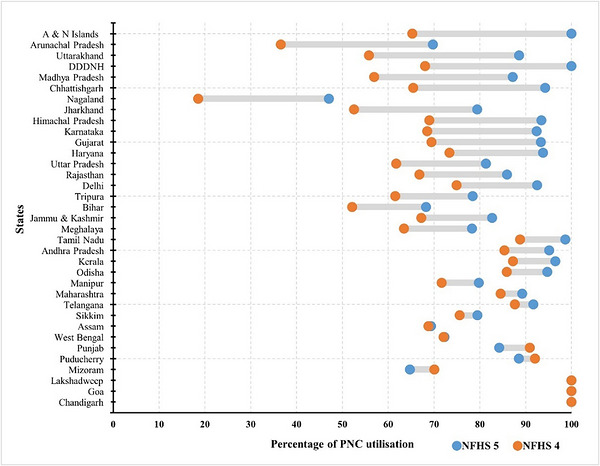
State‐wise comparison of postnatal care (PNC) utilisation among adolescent mothers in India, NFHS‐4 (2015–16) and NFHS‐5 (2019–21). A&N Islands, Andaman and Nicobar Islands; DDDNH, Daman and Diu and Dadra and Nagar Haveli.

By 2019–21, the lowest utilisation rates were primarily concentrated in the north‐eastern states: Nagaland, Mizoram (64.7%), Assam (69.3%), Arunachal Pradesh (69.7%) and Meghalaya (78.3%). Bihar (68.2%) and West Bengal (72.3%) also reported comparatively low utilisation during this period. The highest increases in PNC utilisation among adolescent mothers between 2015–16 and 2019–21 were observed in Arunachal Pradesh (33.2 percentage points), followed by Uttarakhand (32.8 percentage points) and Madhya Pradesh (30.3 percentage points). Conversely, the lowest improvements were recorded in West Bengal (0.2 percentage points), Assam (0.6 percentage points) and Sikkim (3.9 percentage points) (see Figure 2).

PNC utilisation also varied across the background characteristics of adolescent mothers (Table 2). Utilisation was positively associated with both educational attainment and household wealth. Adolescent mothers covered by any form of health insurance reported higher levels of PNC utilisation, 78.1% in 2015–16 and 87.0% in 2019–21, compared to those without coverage. Similarly, higher utilisation was observed among adolescent mothers who received eight or more antenatal care visits, with 82.6% in 2015–16 and 90.7% in 2019–21 reporting PNC use. Place of delivery also played a significant role: Adolescent mothers who delivered in public health facilities reported PNC utilisation rates of 74.2% in 2015–16 and 83.3% in 2019–21, whereas those delivering in private facilities had even higher rates (82.7% and 90.9%, respectively).

**TABLE 2:** Postnatal care utilisation among adolescent mothers within 42 days of delivery by background characteristics, India.

Variable	NFHS‐4 (2015–16)			NFHS‐5 (2019–21)		
	% [95% CI]	*χ* ^2^	*p* value	% [95% CI]	*χ* ^2^	*p* value
**Current marital status**		2.1	0.316		0.1	0.795
Married	69.9 [68.9, 71.0]			82.2 [81.2, 83.2]		
Not currently married	66.3 [58.6, 73.2]			81.4 [74.1, 87.0]		
**Education**		319.9	<0.001		176.7	<0.001
No education	59.1 [56.9, 61.3]			72.3 [69.6, 74.7]		
Primary	65.3 [62.7, 67.8]			79.6 [76.7, 82.2]		
Secondary	73.7 [72.4, 74.9]			84.1 [82.9, 85.2]		
Higher	79.6 [75.2, 83.4]			88.1 [84.2, 91.2]		
**Religion**		70.6	<0.001		10.4	0.088
Hindu	70.7 [69.6, 71.8]			82.6 [81.5, 83.6]		
Muslim	63.4 [60.5, 66.3]			79.6 [76.6, 82.4]		
Others	76.6 [72.2, 80.4]			83.1 [78.5, 86.9]		
**Social group**		34.1	0.001		15.7	0.068
SC	69.2 [67.1, 71.3]			80.3 [78.3, 82.2]		
ST	67.4 [64.8, 69.8]			82.7 [80.3, 84.9]		
OBC	69.2 [67.7, 70.6]			83.4 [82.0, 84.6]		
Others	73.6 [71.2, 75.9]			82.1 [79.3, 84.5]		
**Household wealth**		658.8	<0.001		498.8	<0.001
Poorest	56.9 [54.9, 58.8]			73.1 [71.1, 75.0]		
Poorer	67.2 [65.2, 69.1]			79.8 [77.8, 81.6]		
Middle	76.4 [74.6, 78.2]			85.9 [83.9, 87.6]		
Richer	78.5 [76.0, 80.7]			92.1 [90.3, 93.5]		
Richest	82.4 [79.5, 84.9]			92.4 [89.9, 94.3]		
**Health insurance**		100.1	<0.001		57.5	<0.001
No	68.4 [67.3, 69.5]			81.0 [79.8, 82.1]		
Yes	78.1 [75.5, 80.4]			87.0 [85.1, 88.6]		
**ANC visits**		992.7	<0.001		444.3	<0.001
<4	58.1 [56.7, 59.6]			74.3 [72.7, 75.9]		
4‐7	78.2 [76.5, 79.8]			86.3 [84.8, 87.7]		
≥8	82.5 [80.2, 84.6]			90.7 [88.7, 92.3]		
**Place of delivery**		2200.4	<0.001		930.1	<0.001
Home	33.1 [30.5, 35.6]			52.7 [49.2, 56.1]		
Public facility	74.1 [72.9, 75.4]			83.3 [82.1, 84.4]		
Private facility	82.7 [80.9, 84.3]			90.9 [89.3, 92.3]		
**Sex of the child**		0.8	0.505		0.4	0.654
Male	70.2 [68.8, 71.5]			82.0 [80.7, 83.2]		
Female	69.5 [68.1, 71.0]			82.4 [81.0, 83.8]		
**Birth order**		84.4	<0.001		42.4	<0.001
First	71.6 [70.4, 72.7]			83.1 [82.0, 84.1]		
Second	65.2 [62.9, 67.4]			80.1 [77.8, 82.2]		
Third or more	59.1 [53.2, 64.8]			71.7 [65.2, 77.4]		
**Place of residence**		98.2	<0.001		52.9	<0.001
Urban	76.2 [73.8, 78.4]			86.8 [84.3, 88.9]		
Rural	68.1 [66.9, 69.2]			81.0 [79.9, 82.1]		
**Region of residence**		647.0	<0.001		733.3	<0.001
North	70.2 [67.8, 72.5]			87.4 [85.1, 89.3]		
Central	60.3 [58.5, 62.1]			84.1 [82.3, 85.8]		
East	64.2 [62.2, 66.2]			73.3 [71.3, 75.2]		
North‐east	66.1 [63.0, 69.1]			71.8 [68.7, 74.7]		
West	80.5 [77.5, 83.2]			90.4 [87.5, 92.6]		
South	83.1 [80.9, 85.1]			94.9 [93.5, 95.9]		
**India**	69.9 [68.8, 70.9]			82.2 [81.2, 83.2]		

Abbreviations: 95% CI, 95% confidence interval; ANC, antenatal care; OBC, Other Backward Class; SC, Scheduled Caste; ST, Scheduled Tribe.

In contrast, utilisation remained substantially lower among mothers who delivered at home (33.1% in 2015–16 and 52.7% in 2019–21). PNC utilisation declined with increasing birth order of the child. Urban adolescent mothers consistently reported higher utilisation (76.2% in 2015–16 and 86.8% in 2019–21) compared to their rural counterparts (68.1% and 81.1%, respectively). Regionally, the highest levels of PNC utilisation were recorded in the Southern region (83.2% in 2015–16 and 94.9% in 2019–21), whereas the north‐eastern region reported the lowest (66.2% in 2015–16 and 71.9% in 2019–21).

### 3.3 Determinants of PNC Utilisation Among Adolescent Mothers

Table 3 presents the AORs for factors associated with PNC utilisation among adolescent mothers in India. The results indicate a significant increase in PNC utilisation between the two survey rounds. Compared with 2015–16, adolescent mothers in 2019–21 were 91% more likely to receive PNC within 42 days of delivery (AOR: 1.91, *p* < 0.001), indicating substantial improvement in PNC utilisation over time.

**TABLE 3:** Adjusted odds ratios for factors associated with postnatal care utilisation among adolescent mothers in India, pooled analysis of NFHS‐4 (2015–16) and NFHS‐5 (2019–21).

Variables	AOR (95% CI)	*p* value
**Survey year**		
2015–16	1.00 (Ref.)	
2019–21	1.91 [1.77, 2.07]	<0.001
**Current marital status**		
Married	1.00 (Ref.)	
Not currently married	0.89 [0.66, 1.21]	0.469
**Education**		
No education	1.00 (Ref.)	
Primary	1.00 [0.88, 1.14]	0.989
Secondary	1.03 [0.93, 1.15]	0.511
Higher	1.10 [0.87, 1.38]	0.421
**Religion**		
Hindu	1.00 (Ref.)	
Muslim	0.88 [0.78, 1.00]	0.051
Others	1.30 [1.06, 1.58]	0.012
**Social group**		
Others	1.00 (Ref.)	
SC	0.97 [0.84, 1.12]	0.694
ST	1.12 [0.96, 1.31]	0.148
OBC	0.96 [0.85, 1.09]	0.531
**Household wealth**		
Poorest	1.00 (Ref.)	
Poorer	1.09 [0.98, 1.21]	0.106
Middle	1.35 [1.19, 1.52]	<0.001
Richer	1.47 [1.27, 1.71]	<0.001
Richest	1.64 [1.34, 2.02]	<0.001
**Covered with health insurance**		
No	1.00 (Ref.)	
Yes	1.28 [1.14, 1.44]	<0.001
**ANC visits**		
<4	1.00 (Ref.)	
4–7	1.84 [1.69, 2.02]	<0.001
≥8	2.05 [1.79, 2.36]	<0.001
**Place of delivery**		
Home	1.00 (Ref.)	
Public facility	3.91 [3.52, 4.34]	<0.001
Private facility	5.62 [4.90, 6.44]	<0.001
**Sex of the child**		
Male	1.00 (Ref.)	
Female	0.98 [0.91, 1.06]	0.65
**Birth order of child**		
First	1.00 (Ref.)	
Second	0.89 [0.81, 0.98]	0.015
Third or more	0.83 [0.66, 1.02]	0.081
**Place of residence**		
Urban	1.00 (Ref.)	
Rural	1.01 [0.89, 1.14]	0.937
**Region of residence**		
North	1.00 (Ref.)	
Central	0.97 [0.87, 1.09]	0.646
East	0.79 [0.70, 0.89]	<0.001
North‐east	0.79 [0.68, 0.91]	0.002
West	1.38 [1.15, 1.65]	0.001
South	1.50 [1.28, 1.76]	<0.001

Abbreviations: ANC, antenatal care; AOR, adjusted odds ratio; CI, confidence interval; OBC, Other Backward Class; Ref., reference category; SC, Scheduled Caste; ST, Scheduled Tribe.

Socio‐economic status was an important determinant of PNC utilisation. The likelihood of receiving PNC increased progressively with household wealth. Adolescent mothers from the richest households had 64% higher odds of receiving PNC (AOR: 1.64, *p* < 0.001) compared with those from the poorest households. Health insurance coverage also showed a positive association with PNC utilisation. Adolescent mothers covered by any form of health insurance were 28% more likely to receive PNC than those without insurance coverage (AOR: 1.28, *p* < 0.001).

Antenatal care attendance showed a strong and graded association with PNC utilisation. Compared with mothers who received fewer than four antenatal care visits, those who received four to seven visits were 84% more likely to utilise PNC (AOR: 1.84, *p* < 0.001). The likelihood was even higher among mothers who received eight or more antenatal visits, who had more than twice the odds of receiving PNC (AOR: 2.05, *p* < 0.001). This pattern suggests a clear dose–response relationship between antenatal care utilisation and PNC uptake.

Place of delivery also had a strong influence on PNC utilisation. Adolescent mothers who delivered in public health facilities were nearly four times more likely to receive PNC (AOR: 3.91, *p* < 0.001), whereas those who delivered in private facilities were more than five times as likely to receive PNC (AOR: 5.62, *p* < 0.001), compared with mothers who delivered at home.

Regional differences in PNC utilisation were also evident. Adolescent mothers residing in the eastern (AOR: 0.79, *p* < 0.001) and north‐eastern (AOR: 0.79, *p* = 0.002) regions had significantly lower odds of receiving PNC compared with those in the northern region. In contrast, adolescent mothers in the western (AOR: 1.38, *p* = 0.001) and southern (AOR: 1.50, *p* < 0.001) regions were significantly more likely to receive PNC than those in the north, indicating persistent regional disparities in access to PNC services.

## 4 Discussion

This study provides updated national evidence on PNC utilisation among adolescent mothers in India using data from NFHS‐4 and NFHS‐5. The findings indicate a substantial increase in PNC uptake between the two survey rounds, reflecting the broader progress of maternal health services in India. However, the analysis also reveals persistent socio‐economic and regional inequalities that warrant attention. The results further suggest that household wealth, health insurance coverage, antenatal care visits, place of delivery and region of residence are important factors associated with PNC utilisation among adolescent mothers.

One of the most striking findings of this study is the strong influence of place of delivery on PNC. Adolescent mothers who delivered in public or private health facilities were several times more likely to receive PNC compared to those who delivered at home, with the association being particularly strong for private facilities. It is in line with previous studies [12, 29, 30, 31, 32]. This highlights institutional delivery as a critical gateway to PNC. Facility‐based births increase contact with health professionals, create opportunities for counselling and facilitate follow‐up services, all of which substantially improve the likelihood of receiving PNC [33, 34, 35]. Notably, deliveries in private facilities were linked with even higher odds of PNC use compared to public institutions. This may reflect differences in service organisation, follow‐up practices or perceived quality of care in private facilities. These facilities are often equipped to provide comprehensive services and to manage postnatal complications more effectively. Although institutional delivery initiatives have successfully reduced maternal mortality in India, our findings suggest that they also play a critical role in facilitating access to PNC. Ensuring that facility‐based deliveries are systematically linked with follow‐up care, especially for adolescent mothers, should be a key programmatic priority.

The study also demonstrates a clear dose–response relationship between ANC utilisation and PNC uptake. Adolescent mothers who attended four or more ANC visits, and especially those who received eight or more visits, were significantly more likely to utilise postnatal services. This reinforces earlier evidence from India and other low‐ and middle‐income countries, which consistently shows that regular ANC serves as a critical gateway to continued maternal healthcare, including PNC [12, 29, 30, 31, 32]. This finding underscores the role of ANC not only as a clinical intervention but also as a platform for health education and care continuity. Regular ANC visits may increase awareness of postnatal health needs, improve familiarity with health facilities and strengthen trust in the health system, thereby facilitating subsequent PNC use. These results suggest that efforts to improve PNC utilisation among adolescents should not be isolated from strategies to expand and improve ANC coverage, especially encouraging adolescents to meet or exceed the recommended minimum number of visits [2, 33, 36, 37].

Despite improvements in ANC attendance and institutional delivery, PNC remains the weakest link in the maternal healthcare continuum. Although many adolescent mothers now access services during pregnancy and delivery as a result of continuous efforts such as the *Pradhan Mantri Surakshit Matritva Abhiyan* (focused on ANC) and *Janani Suraksha Yojana* (focused on institutional delivery) [38], a substantial proportion still do not receive timely PNC, particularly beyond the immediate post‐delivery period. This gap indicates that gains achieved earlier in the continuum are not consistently translated into sustained postnatal follow‐up. Existing maternal health programmes in India have prioritised antenatal care and safe delivery, but comparatively less emphasis has been placed on ensuring continuity of care after childbirth. Strengthening postnatal outreach, follow‐up visits and counselling, especially for adolescent mothers, thus remains a critical challenge. Addressing this requires a paradigm shift in maternal health policy, moving from a narrow focus on delivery to a broader life‐course approach that integrates antenatal, intrapartum and PNC seamlessly.

Socio‐economic inequalities continue to shape access to PNC [13, 39]. Adolescent mothers from poorer households [22, 28, 36, 37] and those without health insurance [40, 41] were significantly less likely to utilise PNC services, even after adjusting for other factors. These findings highlight persistent financial and structural barriers to care, including indirect costs such as transportation and loss of wages, which disproportionately affect adolescents who often lack financial autonomy. Although schemes such as Janani Suraksha Yojana and Ayushman Bharat aim to reduce financial barriers [33, 42, 43], the results suggest that adolescents may not be fully benefiting from these initiatives, underscoring the need for more targeted financial protection and outreach.

Regional disparities were also pronounced, with adolescent mothers in the north‐eastern and eastern regions exhibiting consistently lower PNC utilisation. These patterns likely reflect a combination of geographic isolation, limited health infrastructure, cultural practices and weaker health system connectivity [44]. The persistence of these regional gaps, even as national coverage improves, indicates that uniform national strategies may be insufficient. Context‐specific, regionally tailored interventions are required to address structural barriers and improve postnatal service delivery in underserved areas.

Taken together, these findings indicate that recent improvements in maternal healthcare in India have not translated evenly into PNC coverage for adolescent mothers. While expanded antenatal care and institutional delivery have created important entry points into the health system, the transition from delivery to sustained postnatal follow‐up remains weak. The strong associations observed for antenatal care utilisation and place of delivery suggest that PNC uptake is shaped less by isolated postnatal interventions and more by continuity across the maternal health pathway. However, this continuity is disrupted by socio‐economic disadvantage, lack of insurance coverage and regional health system constraints, particularly in underserved areas. In addition, some social and cultural factors also influence PNC use. In many communities, women have limited mobility after childbirth and may depend on male family members for transportation or permission to visit health facilities, which can delay or prevent care‐seeking. In addition, traditional practices often require mothers to remain inside the home for about 30–40 days after delivery. Although these practices are intended to protect the mother and newborn, they can reduce contact with health services and make it more difficult for mothers to attend postnatal check‐ups or receive visits from health workers [45, 46]. Improving PNC for adolescent mothers therefore requires more than simply increasing service coverage. It requires strengthening continuity between antenatal care, delivery services and postnatal follow‐up while also addressing the social and cultural conditions that shape healthcare use among vulnerable groups and regions.

A key strength of this study is the use of large, nationally representative data, which allow for a detailed examination of PNC utilisation among adolescent mothers, a group that remains under‐represented in maternal health research. Pooling data from NFHS‐4 and NFHS‐5 enabled the assessment of changes over time and the identification of persistent socio‐economic and regional disparities.

Nevertheless, several limitations should be acknowledged. First, the analysis relies on self‐reported information, which may be subject to recall and social desirability bias, particularly in reporting PNC utilisation. Second, the cross‐sectional design of the NFHS precludes causal inference, as unobserved factors may influence both antenatal care use and PNC uptake. Third, the outcome variable captures only whether a postnatal check occurred within the specified timeframe and does not include detailed information on the precise timing of the visit, the number of visits received, the type of provider consulted or the clinical quality and content of care delivered. Consequently, our findings reflect contact with postnatal services rather than the adequacy, continuity or comprehensiveness of postpartum care. In addition, some explanatory variables, such as household wealth and health insurance coverage, are measured at the time of the survey and may not fully capture the socio‐economic circumstances at the time of delivery. Finally, broader contextual and cultural determinants, such as health system accessibility, intra‐household decision‐making dynamics and local service availability, are not fully measured in survey data. Future research using longitudinal, mixed‐methods or qualitative approaches could provide deeper insights into the quality and lived experience of PNC among adolescent mothers.

## 5 Conclusion

In conclusion, PNC utilisation among adolescent mothers in India has increased substantially in recent years, reflecting broader improvements in maternal health service coverage. However, important socio‐economic, regional and service‐related inequalities persist. Adolescent mothers who are poor, uninsured, have limited antenatal care exposure, deliver at home or live in underserved regions remain significantly less likely to receive PNC. Despite progress in antenatal care and institutional delivery, PNC continues to represent a weak link in the maternal health continuum. Strengthening continuity between antenatal care, facility‐based delivery and postnatal follow‐up, while addressing persistent socio‐economic and regional disparities, will be essential for improving equitable access to postnatal services among adolescent mothers.

## Author Contributions

**Rakesh Chandra:** conceptualisation, methodology, validation, writing – original draft, writing – review and editing, project administration. **Muhammad Hossain:** conceptualisation, methodology, writing – original draft, writing – review and editing, investigation, validation, supervision, funding acquisition. **Utkarsh Jain:** writing – review and editing, writing – original draft, data curation. **Anshika Singh:** data curation, investigation, formal analysis, visualisation, writing – original draft, writing – review and editing, methodology, software, validation. **Aditya Singh:** conceptualisation, methodology, formal analysis, visualisation, investigation, validation, supervision, writing – original draft, writing – review and editing.

## Funding

The authors have nothing to report.

## Ethics Statement

This study is based on secondary analysis of data from the National Family Health Survey (NFHS‐4 and NFHS‐5), conducted by the International Institute for Population Sciences (IIPS), Mumbai, in collaboration with ICF. The NFHS surveys received ethical approval from the Institutional Review Board of IIPS, Mumbai, as well as from the ICF Institutional Review Board. As this study involved secondary analysis of anonymised data with no direct participant contact, additional ethical approval was not required.

## Consent

Written informed consent was obtained from all participants prior to data collection. The datasets are de‐identified and publicly available upon request from the DHS/NFHS data archive.

## Conflicts of Interest

The authors declare no conflicts of interest.

## Data Availability

The dataset analysed during the current study is available at https://www.iipsdata.ac.in/major_studies/5/project_details and can be obtained for free by sending an online request.

## References

[1] World Health Organization , “Postnatal Care of the Mother and Newborn,” in Counselling for Maternal and Newborn Health Care: A Handbook for Building Skills (World Health Organization, 2013).

[2] S. Dhakal , G. N. Chapman , P. P. Simkhada , E. R. van Teijlingen , J. Stephens , and A. E. Raja , “Utilisation of Postnatal Care Among Rural Women in Nepal,” BMC Pregnancy Childbirth 7 (2007): 1–9, 10.1186/1471-2393-7-19. 17767710 PMC2075509

[3] D. Mohan , S. Gupta , A. LeFevre , E. Bazant , J. Killewo , and A. H. Baqui , “Determinants of Postnatal Care Use at Health Facilities in Rural Tanzania: Multilevel Analysis of a Household Survey,” BMC Pregnancy Childbirth 15, no. 1 (2015): 1–10, 10.1186/s12884-015-0717-7. 26518337 PMC4628262

[4] D. Javadi , E. Sacks , V. Brizuela , et al., “Factors That Influence the Uptake of Postnatal Care Among Adolescent Girls: A Qualitative Evidence Synthesis,” BMJ Global Health 8, no. S2 (2023): e011560, 10.1136/bmjgh-2022-011560. PMC10163540 37137533

[5] G. Taneja , V. S. R. Sridhar , J. S. Mohanty , et al., “India's RMNCH+A Strategy: Approach, Learnings and Limitations,” BMJ Global Health 4, no. 3 (2019): 1–12. 10.1136/bmjgh-2018-001162 PMC6509590 31139464

[6] World Health Organization , Adolescent Health (World Health Organization, 2024), https://www.who.int/health‐topics/adolescent‐health#tab=tab_1.

[7] C. Sivagurunathan , R. Umadevi , R. Rama , and S. Gopalakrishnan , “Adolescent Health: Present Status and Its Related Programmes in India. Are We in the Right Direction?” Journal of Clinical and Diagnostic Research 9, no. 3 (2015): LE01–6. 10.7860/JCDR/2015/11199.5649 PMC4413087 25964884

[8] IIPS and ICF , National Family Health Survey (NFHS‐5), 2019–21: India. Mumbai (IIPS and ICF , 2021).

[9] S. Youseflu , S. Kohan , and F. Mostafavi , “Promoting Adolescent Mother Self‐Efficacy for Parenting Roles, and Self‐Care After Childbirth: Protocol for a Mixed Methods Study,” Reproductive Health 20, no. 1 (2023): 1–9, 10.1186/s12978-023-01679-9. PMC10548586 37794372

[10] World Health Organization , Adolescent Pregnancy (World Health Organization, 2024), https://www.who.int/news‐room/fact‐sheets/detail/adolescent‐pregnancy.

[11] C. Meh , A. Sharma , U. Ram , et al., “Trends in Maternal Mortality in India Over Two Decades in Nationally Representative Surveys,” BJOG: An International Journal of Obstetrics & Gynaecology 129, no. 4 (2022): 550–561, 10.1111/1471-0528.16888. 34455679 PMC9292773

[12] P. Paul , “Geographical Variations in Postnatal Care Use and Associated Factors in India: Evidence From a Cross‐Sectional National Survey,” Geography Journal 87, no. 1 (2022): 21–34, 10.1007/s10708-020-10241-0.

[13] S. Jung , H. Chi , Y. J. Eom , S. V. Subramanian , and R. Kim , “Multilevel Analysis of Determinants in Postnatal Care Utilisation Among Mother‐Newborn Pairs in India, 2019–21,” Journal of Global Health 14 (2024): 1–15, 10.7189/jogh.14.04085. PMC11079700 38721673

[14] S. Gandhi , S. Gandhi , U. Dash , and M. Suresh Babu , “Predictors of the Utilisation of Continuum of Maternal Health Care Services in India,” BMC Health Services Research 22, no. 1 (2022): 1–12, 10.1186/s12913-022-07876-9. 35513830 PMC9069727

[15] A. Kothavale and T. Meher , “Level of Completion Along Continuum of Care for Maternal, Newborn and Child Health Services and Factors Associated With It Among Women in India: A Population‐Based Cross‐Sectional Study,” BMC Pregnancy Childbirth 21, no. 1 (2021): 1–12, 10.1186/s12884-021-04198-2. 34706680 PMC8554854

[16] A. Baten , R. K. Biswas , E. Kendal , and J. Bhowmik , “Utilization of Maternal Healthcare Services in Low‐ and Middle‐Income Countries: A Systematic Review and Meta‐Analysis,” Systematic Reviews 14, no. 1 (2025): 88, 10.1186/s13643-025-02832-0. 40241227 PMC12004674

[17] S. Aziz , A. Basit , S. Sultana , C. S. E. Homer , and J. P. Vogel , “Inequalities in Women's Utilization of Postnatal Care Services in Bangladesh From 2004 to 2017,” Scientific Reports 12, no. 1 (2022): 1–11, 10.1038/s41598-022-06672-z. 35177728 PMC8854580

[18] S. Gandhi , U. Dash , and M. Suresh Babu , “Horizontal Inequity in the Utilisation of Continuum of Maternal Health Care Services (CMHS) in India: An Investigation of Ten Years of National Rural Health Mission (NRHM),” International Journal for Equity in Health 21, no. 1 (2022): 7, 10.1186/s12939-021-01602-3. 35033087 PMC8760767

[19] P. Singh , K. K. Singh , and P. Singh , “Maternal Health Care Service Utilization Among Young Married Women in India, 1992–2016: Trends and Determinants,” BMC Pregnancy Childbirth 21, no. 1 (2021): 1–13, 10.1186/s12884-021-03607-w. 33568078 PMC7877063

[20] B. S. Mehta , R. Alambusha , A. Misra , N. Mehta , and A. Madan , “Assessment of Utilisation of Government Programmes and Services by Pregnant Women in India,” PLoS ONE 18 (2023): 1–19, 10.1371/journal.pone.0285715. PMC10553210 37796937

[21] B. Ali , P. Debnath , and T. Anwar , “Inequalities in Utilisation of Maternal Health Services in Urban India: Evidences From National Family Health Survey‐4,” Clinical Epidemiology and Global Health 10 (2021): 100672, 10.1016/j.cegh.2020.11.005.

[22] A. Singh , S. S. Padmadas , U. S. Mishra , S. Pallikadavath , F. A. Johnson , and Z. Matthews , “Socio‐Economic Inequalities in the Use of Postnatal Care in India,” PLoS ONE 7, no. 5 (2012): e37037, 10.1371/journal.pone.0037037. 22623976 PMC3356397

[23] P. Tripathi , M. Chakrabarty , A. Singh , and S. Let , “Geographic Disparities and Determinants of Full Utilization of the Continuum of Maternal and Newborn Healthcare Services in Rural India,” BMC Public Health [Electronic Resource] 24, no. 1 (2024): 3378, 10.1186/s12889-024-20714-3. 39639301 PMC11619281

[24] M. Usman , U. S. Reddy , L. A. Siddiqui , and A. Banerjee , “Exploration of Spatial Clustering in Maternal Health Continuum of Care Across Districts of India: A Geospatial Analysis of Demographic and Health Survey Data,” PLoS ONE 17 (2022): 1–17, 10.1371/journal.pone.0279117. PMC9754170 36520872

[25] Y. Krishnamoorthy and M. G. Majella , “Determinants of Postnatal Care Coverage Among Mothers and New‐Borns in India: Evidence From a Nationally Representative Survey,” International Journal of Health Planning and Management 36, no. 4 (2021): 1276–1286, 10.1002/hpm.3179. 33866592

[26] P. Barman , N. Sarif , and A. Saha , “Association Between Natural Hazards and Postnatal Care Among the Neonates in India: A Step Towards Full Coverage Using Geospatial Approach,” BMC Emergency Medicine 23, no. 1 (2023): 1–13, 10.1186/s12873-023-00844-4. 37460972 PMC10351138

[27] International Institute for Population Sciences (IIPS) and ICF , National Family Health Survey (NFHS‐4) (International Institute for Population Sciences (IIPS) and ICF, 2017), http://www.rchiips.org/nfhs%0Ahttp://rchiips.org/NFHS/NFHS‐4Reports/India.pdf.

[28] N. Chakraborty , M. A. Islam , R. I. Chowdhury , and W. Bari , “Utilisation of Postnatal Care in Bangladesh: Evidence From a Longitudinal Study,” Heal Care Community 10, no. 6 (2002): 492–502, 10.1046/j.1365-2524.2002.00389.x. 12485137

[29] V. Khanal , M. Adhikari , R. Karkee , and T. Gavidia , “Factors Associated With the Utilisation of Postnatal Care Services Among the Mothers of Nepal: Analysis of Nepal Demographic and Health Survey 2011,” BMC Women Health 14, no. 1 (2014): 19, 10.1186/1472-6874-14-19. PMC3911793 24484933

[30] D. Akunga , D. Menya , and M. Kabue , “Determinants of Postnatal Care Use in Kenya,” African Population Studies 28, no. 3 (2014): 1447–1459.

[31] T. Dahiru and O. M. Oche , “Determinants of Antenatal Care, Institutional Delivery and Postnatal Care Services Utilization in Nigeria,” Pan African Medical Journal 21 (2015): 1–17, 10.11604/pamj.2015.21.321.6527. 26587168 PMC4633744

[32] A. Singh , A. Yadav , and A. Singh , “Utilization of Postnatal Care for Newborns and Its Association With Neonatal Mortality in India: An Analytical Appraisal,” BMC Pregnancy Childbirth 12, no. 33 (2012): 2–7, https://bmcpregnancychildbirth.biomedcentral.com/track/pdf/10.1186/1471‐2393‐12‐33. 22571689 10.1186/1471-2393-12-33 PMC3405468

[33] N. Daliana , N. Farid , N. M. Azahar , K. Chinna , F. N. Seraz , and O. M. Oche , “Factors Associated With the Utilization of Postnatal Care Services Among Adolescent Mothers in Nigeria,” African Journal of Nursing and Midwifery 10, no. 8 (2022): 1–12.

[34] D. Nuryana , P. Viwattanakulvanid , and N. A. Romadlona , “Maternal Health Services Utilization and Its Contributing Factors Among Adolescent Mothers,” International Journal of Public Health Science (IJPHS) 11, no. 1 (2022): 77–87, 10.11591/ijphs.v11i1.21041.

[35] C. Chungu , M. Makasa , M. Chola , and C. N. Jacobs , “Place of Delivery Associated With Postnatal Care Utilization Among Childbearing Women in Zambia,” Frontiers in Public Health 6 (2018): 1–7. 29682497 10.3389/fpubh.2018.00094 PMC5897430

[36] A. B. Geremew , M. M. Boke , and A. E. Yismaw , “The Effect of Antenatal Care Service Utilization on Postnatal Care Service Utilization: A Systematic Review and Meta‐Analysis Study,” Journal of Pregnancy 2020 (2020): 7363242, 10.1155/2020/7363242. 33029402 PMC7528140

[37] O. E. Banke‐Thomas , A. O. Banke‐Thomas , and C. A. Ameh , “Factors Influencing Utilisation of Maternal Health Services by Adolescent Mothers in Low‐and Middle‐Income Countries: A Systematic Review,” BMC Pregnancy Childbirth 17, no. 1 (2017): 1–14, 10.1186/s12884-017-1246-3. 28209120 PMC5314631

[38] N. Carvalho and S. Rokicki , “The Impact of India's Janani Suraksha Yojana Conditional Cash Transfer Programme: A Replication Study,” Journal of Development Studies 55, no. 5 (2019): 989–1006, 10.1080/00220388.2018.1506578.

[39] P. S. Mukonka , P. K. Mukwato , C. N. Kwaleyela , and O. Mweemba , “Household Factors Associated With Use of Postnatal Care Services,” African Journal of Midwifery and Women's Health 12, no. 4 (2018): 189–193, 10.12968/ajmw.2018.12.4.189.

[40] I. Ali , S. N. Akhtar , B. G. Chauhan , M. A. Malik , and K. D. Singh , “Health Insurance Support on Maternal Health Care: Evidence From Survey Data in India,” Journal of Public Health (Oxford) 45, no. 2 (2022): 368–378, 10.1093/pubmed/fdac025. 35285932

[41] T. A. Gebremedhin , I. Mohanty , and T. Niyonsenga , “Public Health Insurance and Maternal Health Care Utilization in India: Evidence From the 2005–2012 Mothers' Cohort Data,” BMC Pregnancy Childbirth 22 (2022): 155, 10.1186/s12884-022-04441-4. 35216564 PMC8876067

[42] A. D. Wuneh , A. A. Medhanyie , A. M. Bezabih , L. Å. Persson , J. Schellenberg , and Y. B. Okwaraji , “Wealth‐Based Equity in Maternal, Neonatal, and Child Health Services Utilization: A Cross‐Sectional Study From Ethiopia,” International Journal for Equity in Health 18, no. 1 (2019): 1–9, 10.1186/s12939-019-1111-2. 31870447 PMC6929360

[43] R. Ambarish Kumar , “Inequality in Utilization of Maternal Health Care Services Among Teenage Married Women in Uttar Pradesh: Evidence From NFHS‐3,” Global Journal of Multidisciplinary Studies 3, no. 10 (2014).

[44] G. Kumar and R. S. Reshmi , “Availability of Public Health Facilities and Utilization of Maternal and Child Health Services in Districts of India,” Clinical Epidemiology and Global Health 15 (2022): 101070, 10.1016/j.cegh.2022.101070.

[45] R. K. Dehury , A. Pati , and P. Dehury , “Traditional Practices and Beliefs in Post‐Partum Care: Tribal Women in Maharashtra,” ANTYAJAA: Indian Journal of Women and Social Change 3, no. 1 (2018): 49–63, 10.1177/2455632718778391.

[46] A. Sarkar , O. M. Kharmujai , W. Lynrah , and N. U. Suokhrie , “Factors Influencing the Place of Delivery in Rural Meghalaya, India: A Qualitative Study,” Journal of Family Medicine and Primary Care 7, no. 1 (2018): 98–103, https://journals.lww.com/jfmpc/fulltext/2018/07010/factors_influencing_the_place_of_delivery_in_rural.18.aspx. 10.4103/jfmpc.jfmpc_45_17 PMC5958601 29915741

